# A Hat-Pin Swallowed by an Infant

**Published:** 1901-05-25

**Authors:** 


### A HAT-PIN SWALLOWED BY AN INFANT.

A curious case is reported from St. Thomas's Hospital under the care of Mr. Ballance.[1] The patient was an infant aged 15 months, who, 18 hours before admission, while standing on a chair had placed a lady's hat-pin (six inches long) headforemost in his mouth. He fell from the chair, and the pin, probably from striking the ground, was pushed down his throat. His mother could see the point, but in trying to pull it out only pushed it further down. The child subsequently swallowed two pieces of toast without any difficulty, and when brought to the hospital showed no signs of anything being amiss. Skiagrams, however, proved the pin to be in the lower end of the œsophagus, the head being about the level of the cardiac end of the stomach. Thirty hours after the accident the stomach was opened, the œsophagus having first been explored by the finger without result. The stomach was exposed and held up to the surface by two threads passed into its wall, and a small incision was made between the threads. On the finger being introduced the head of the pin was felt presenting at the cardiac opening, and it was extracted without difficulty. For 24 hours the child was fed *per rectum.* He recovered perfectly. Some time ago a case was recorded in which a similar accident happened to an adult. A woman, thinking that something she was eating had stuck in her throat, tried to ram it down with her hat-pin. In so doing the point got jammed behind her upper incisor teeth, and while she was endeavouring to dislodge it the pin slipped from her fingers and was swallowed. In this case the whole of the pin entered into the stomach and at the operation the surgeon, instead of incising the stomach wall, pushed the point through until the head prevented its further passage and then, cutting the pin off short with wire nippers, left the head to pass away *per vias naturales,* which it did.

[1] Clinical Journal, May 15.

